# Thermal degradation behavior and flame retardant properties of PET/DiDOPO conjugated flame retardant composites*

**DOI:** 10.3389/fchem.2022.1018998

**Published:** 2022-10-07

**Authors:** Yushu Xiang, Yun Gao, Guomin Xu, Min He, Shuhao Qin, Jie Yu

**Affiliations:** ^1^ College of Materials and Metallurgy, Guizhou University, Guiyang, China; ^2^ National Engineering Research Center for Compounding and Modification of Polymer Materials, Guiyang, China

**Keywords:** DOPO derivative, PET, flame retardant, rheological behavior, thermal degradation behavior

## Abstract

PET/DIDOPO conjugated flame retardant composites were prepared by melt blending of styrene bridged DOPO (DIDOPO) into polyethylene terephthalate (PET). The flame retardancy, rheological behavior, and thermal degradation behavior of the composite were characterized by vertical combustion test (UL-94), limit oxygen index test (LOI), rotational rheometer, and thermogravimetry (TG). The results showed that the flame retardant composite with V-0 grade was obtained when the amount of DIDOPO is 12.5wt%, and the corresponding LOI value was 56.87% higher than that of PET. The thermogravimetry-fourier infrared spectroscopy (TG-FTIR) test results showed that DIDOPO could promote the degradation of PET/DIDOPO materials, and release phosphorus-containing free radicals to quench the flame, therefore slowing down the combustion process, and mainly playing the key flame retardant role in gas-phase.

## 1 Introduction

As a semi-crystalline thermoplastic polyester material, polyethylene terephthalate (PET) is widely used in automobiles, electronics, and machinery. However, the flame retardancy of PET is relatively poor, and its limiting oxygen index is only about 21%, accompanied by serious droplet phenomenon, which greatly limits the application of PET in the field with requirements for combustion safety. Therefore, the flame retardant modification of PET materials becomes particularly important (Vannier et al., 2008).

In recent years, phosphorous-containing compounds have attracted wide attention for being halogen-free and having high efficiency in the fire protection of polymers, such as 9,10-dihydro-9-oxa-10-phosphaphenanthrene-10-oxide (DOPO) and its derivatives (Perez et al., 2006; Zhang et al., 2011; Zhang et al., 2022a; Zhang et al., 2022b), hypophosphite (Ge et al., 2015), metal phosphinate (Zhao et al., 2013; He et al., 2020), etc., (Si et al., 2014). Among these flame retardants, the most important and efficient flame retardants are metal phosphinates, which can play their main role in gas phase by releasing PO·free radical scavenger and also catalyze the charring in the condensed phase. Higher than 15 wt% loading of metal phosphinates is generally necessary to pass the UL-94 tests, but the large addition amounts will unavoidable decrease the mechanical properties (Zhao et al., 2013; Lin et al., 2018). DOPO and its modifications, as a new class of the additive-type flame retardants, have received outstanding attention due to their high thermal stability and flame retardancy. Buczko et al. (2014) synthesized two bridged DOPO derivatives and added them to PA6. The results showed that maximum decomposition temperatures (T_max_) of DiDOPO-MeO containing P-O bonds and DiDOPO-EDA containing P-N bonds were 422°C and 384°C, respectively. The thermal stability of the material decreases after compounding into PA6, while the char residue rate increases significantly. When the addition amount of flame retardant was 17 wt%, the flame retardant composite material could pass the UL-94 V-0 level, and the total heat release is reduced. Compared with DOPO derivatives containing P-O and P-N bonds, bridged DOPO derivatives containing P-C bonds have better resistance to nucleophilic attack because the carbon substituent is not easy to leave (Butnaru et al., 2015; Wendels et al., 2017). Long et al. (2017) synthesized ethyl-bridged DOPO and phenethyl-bridged DOPO (DIDOPO) with the T_max_ of 498°C and 429°C, respectively, and used them as flame retardants for polylactic acid (PLA) materials. The research results showed that when the addition amount of flame retardant is 10 wt%, a composite material with a flame retardant grade of V-0 was obtained. When the ethyl-bridged DOPO acts as the flame retardant, the flame retardant effect is mainly enhanced by gas-phase flame retardant and the heat removal of molten droplets. Comparably, due to the introduction of the aromatic ring groups, DIDOPO can form a cross-linked structure with PLA to suppress the droplet, and it can improve the compactness of the char layer and lead to a better solid-phase flame retardant effect. Compared with metal hypophosphonate, bridge DOPO derivatives have higher thermal stability and better charring effect. At similar flame retardant loading, the bridged DOPO derivatives show better compatibility with the matrix and superior mechanical properties for glass-fiber-reinforced polyamide 6T (Huang et al., 2018). Therefore, bridged DOPO derivatives are expected to be effective flame retardants for PET. However, there is still lack of researches on the flame retardancy and thermal behavior of DIDODP in PET.

Therefore, in this study, DIDOPO with conjugated structure have been added to PET materials with different percentages and the PET/DIDOPO conjugated flame retardant composites are prepared by melt blending. The flame retardant properties, rheological behavior, and thermal degradation behavior of PET/DIDOPO composites have been also studied.

## 2 Materials and methods

### 2.1 Materials

Polyethylene terephthalate (PET) slices (SD500, intrinsic viscosity coefficient 0.68 dl/g): Sinopec Yizheng Chemical Fiber Co., Ltd.; Phenethyl bridged DOPO derivative (DIDOPO) was obtained from our laboratory (Long et al., 2017). Briefly, DOPO (2.0 mol), acetophenone (1.0 mol) and 200 ml xylene were mixed together in a four necked flask equipped, and the reaction was prepared under nitrogen atmosphere. When the temperature of the mixed system reached 180°C, phosphorus oxychloride (0.35 mol) was slowly added in 25 h, and then began to cool down and recrystallize. After standing and filtering, washing with water and drying *in vacuo*, white solid powder was obtained.

### 2.2 Sample preparation

After the flame retardant DIDOPO with conjugated structure and PET pellets were vacuum dried in an oven at 100°C and 130°C for 4 h, respectively, DIDOPO and PET were mixed with weight ratio of 0:100,7.5:92.5, 10:90, 12.5:87.5, 15:85. After mixing, it is extruded and cut through a twin-screw extruder. The temperature of each section of the extruder is: 180°C in zone I, 200°C in zone II, 230°C in zone III, 230°C in zone IV, 240°C in zone V, and head 242°C, with the screw speed 250 r/min, and the feeding speed 18 r/min. Then, the pellets obtained by extrusion and pelletizing were first baked at 100°C for 4 h, and then at 130°C for 4 h. Standard splines were formed by a micro-injection molding machine. Zone III is 246°C, zone IV is 257°C, the head is 257°C, injection is 263°C, and the template zone is 68°C.

### 2.3 Characterization methods

LOI data were acquired by a JF-34 oxygen index (Jiangning, China) according to ASTM D2863-97, and the dimensions of specimens were 100 mm × 6.5 mm × 3.2 mm.

The UL-94 vertical burning ratings were assessed using an SH5302 instrument (Guangzhou, China) according to ASTMD3801 with a three-dimensional size of 125 mm × 13 mm × 3.2 mm.

The rheological behaviors of pure PET and the flame-retardant composites were analyzed using a Rheometric analyzer (HAAKE MARS 40, Thermo Fisher Scientific Inc.) using parallel plates with diameters of 35 mm. At 260°C using 1% strain in an angular frequency ranging from 0.01 to 100 rad/s.

Thermogravimetric analysis (TGA) experiments were performed using a Q50 thermal gravimetric analyzer made by TA Co., Ltd. United States . Apparatus with a nitrogen flow of 60 ml/min. Samples (about 5 mg) were heated in alumina pans, from 25 to 700°C at heating rates of 10°C/min.

## 3 Results and discussion

### 3.1 Analysis of flame retardant properties of PET/DIDOPO conjugated flame retardant composites

**FIGURE 1:**
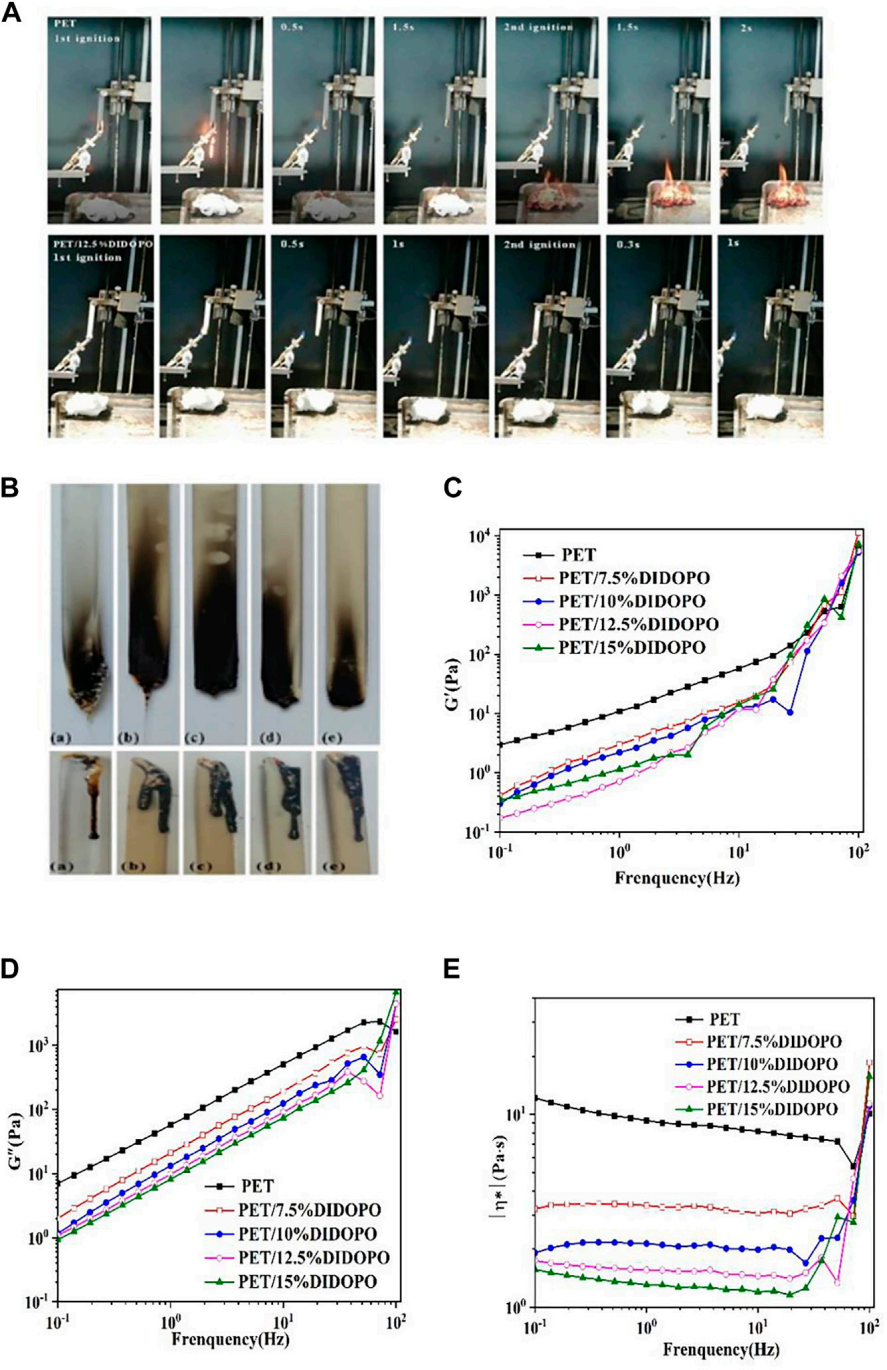
**(A)** UL-94 test process of PET/DIDOPO conjugated flame retardant composite; **(B)** The digital photoshops of the PET/DIDOPO samples after UL-94 tests and LOI test; Evolution of storage modulus **(C)**, loss modulus **(D)**, and complex viscosity **(E)** as a function of frequency for PET/DIDOPO composites.

To evaluate the flame retardant properties of PET/DIDOPO conjugated flame retardant composites, the UL-94 test and the LOI test were performed. Table 1 lists the UL-94 test grades and LOI test results of PET and its flame-retardant composite materials. Figure 1A is the UL-94 test process diagram, and Figure 1B is the spline picture after UL-94 and LOI tests. From the data in the Table and the UL-94 test process diagram, it can be seen that the LOI value of pure PET is only 21.4%. A large number of flame droplets are generated during combustion, and the droplets ignite the absorbent cotton. However, the flame and heat are taken away by the droplets and the spline no longer burns after severe dripping, with a low t1, t2 values and the UL-94 level of V-2. For PET/DIDOPO conjugated flame retardant composites, with the increase of DIDOPO content, the LOI value gradually increases, while (t1 + t2) decreases. When the addition amount increases to 12.5wt% and 15wt%, the absorbent cotton is not ignited though there are molten droplets. The flame retardant grade of the composite material can pass the V-0 level, and the LOI of the flame retardant composite material reaches 33.4%, which is 56.87% higher than that of PET. It can be seen from the spline diagram of the LOI test that the amount of droplets on the surface of the spline increases after DIDOPO with conjugated structure is added. The droplets will take away part of the heat and flame, slowing down the combustion. At the same time, a thin char layer covering the sample was formed, which can protect the underlying matrix.

**TABLE 1:** The UL-94 vertical burning and LOI test results of PET/DIDOPO conjugated flame retardant composites.

Samples	UL-94					
	t_1_ ^a^	t_2_ ^b^	Dripping	Cotton ignition	Rating	LOI
PET	1.4	1.2	Yes	Yes	V-2	21.4 ± 0.2
PET/7.5wt%DIDOPO	0.4	0.6	Yes	Yes	V-2	30.8 ± 0.2
PET/10wt%DIDOPO	0.3	0.2	Yes	Yes	V-2	32.4 ± 0.2
PET/12.5wt%DIDOPO	0.3	0.1	Yes	No	V-0	33.4 ± 0.2
PET/15wt%DIDOPO	0.2	0.2	Yes	No	V-0	33.8 ± 0.2

a t_1_, average combustion times after the first application of the flame.

b t_2_, average combustion times after the second application of the flame.

### 3.2 Analysis of rheological behavior of PET/DIDOPO conjugated flame retardant composites

The droplet phenomenon of polymer materials can be regarded as the flow behavior of polymer melts under the action of gravity (Zhang et al., 2014; Zhao et al., 2014). Therefore, to study the droplet phenomenon of PET/DIDOPO conjugated flame retardant composites, rheological tests have been carried out on PET and its conjugated-flame-retardant composites. Figure 1 shows the relationship between the storage modulus (Gˊ) (C), loss modulus (G″) (D), and complex viscosity (η*) (E) as a function of frequency for PET and PET/DIDOPO conjugated flame retardant composites at 260°C. It can be seen from Figure 1C, Figure 1D that Gˊ, G″ of PET and PET/DIDOPO conjugated flame retardant composites increase with the increase of frequency. The deformation relaxation effect is weakened, and the energy to overcome the intermolecular slip loss per unit time increases, leading to the increase of Gˊ and G". At the same time, like the shear thinning phenomenon that exists in most polymer materials, the viscosity decreases with the increase in frequency, as shown in Figure 1E. By comparison, it is found that at the same frequency, the Gˊ, G″ and η* of PET/DIDOPO conjugated flame retardant composites are lower than that of pure PET, and the higher addition amounts, the lower η* values. The result reveals that DIDOPO with a conjugated structure can act as a plasticizer in PET, which increases the free volume of the flame retardant composite melt, reducing the viscosity and intensifying the phenomenon of molten droplets. These molten droplets take away the flame and heat and slow down the combustion of the material (Li et al., 2014), which is consistent with the flame retardant test results above.

**FIGURE 2:**
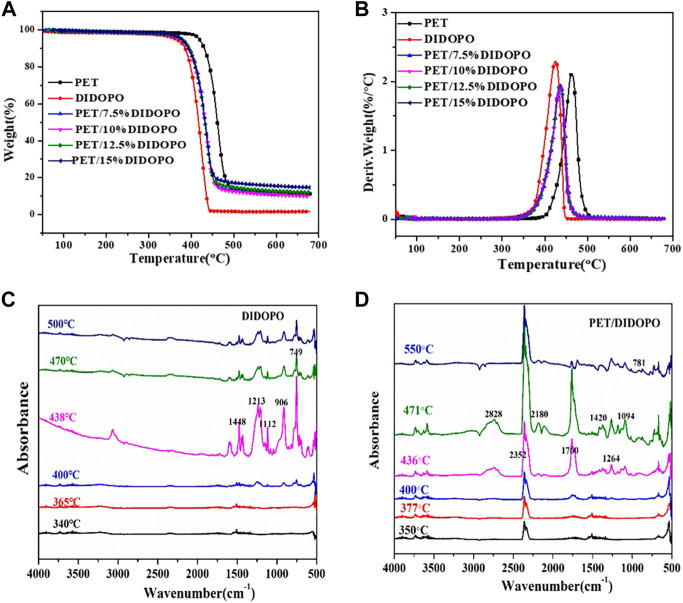
TG **(A)** and DTG **(B)** curves of PET and PET/DIDOPO composites of PET/DIDOPO composites and TG-IR of DIDOPO **(C)** and PET/15wt% DIDOPO flame retardant composites **(D)** at different temperatures spectrum.

### 3.3 Analysis of thermal degradation behavior of PET/DIDOPO conjugated flame retardant composites

TG can determine the thermal degradation temperature of flame retardant composites quickly and reliably. Figures 2A,B are graphs of TG and DTG for PET, DIDOPO with conjugated structure, and PET/DIDOPO conjugated flame retardant composites, and the corresponding the initial decomposition temperature (T_5%_), maximum decomposition temperatures (T_max_), and the char residue at 700°C are listed in Table 2. The thermal degradation process of PET, DIDOPO, and PET/DIDOPO presents one-step degradation, which indicates that the addition of DIDOPO will not change the degradation form of PET. According to our previous work, the T5% of DOPO is estimated at 249°C, and the Tmax is at 328°C (Wang et al., 2021). From Figure 2 and Table 2, the T_5%_ and T_max_ of pure PET are 418.78°C and 463.52°C, respectively, while the T_5%_ and T_max_ of DIDOPO are 364.98°C and 425.28°C, respectively. DIDOPO flame retardants exhibit better thermal stability compared to DOPO. With the addition of DIDOPO in PET, the TG curve shifted to lower temperature, and T_5%_ decreased by about 40°C, and T_max_ decreased by about 26°C, which can be attributed to the degradation of DIDOPO before PET and promote the degradation of PET (Schartel et al., 2007). Comparing the residues at 700°C, it is found that with the increase of the addition amount, the amount of residual char gradually increased, and DIDOPO with conjugated structure had a certain role in promoting carbonization. It is considered that PxOy compounds will be formed based on the reaction of phosphorus and oxygen, and these PxOy compounds will further generate phosphoric acids with H_2_O, which can promote the formation of surface protective char layers, thus play a flame retardant role in condensed-phase (Ramani et al., 2010; Cao et al., 2022a; Lu et al., 2022; Mu et al., 2022). This is consistent with the phenomenon that there is a small amount of char layer on the surface of the PET/DIDOPO conjugated flame retardant composite after burning in the UL-94 tests.

**TABLE 2:** The TG and DTG date of PET and PET composites.

Samples	T_5%_ (°C)	T_max_ (°C)	Residue at 700°C (%)	Theoretical residue at 700°C (%)	Δr (%)
PET	418.78	463.52	11.24	—	—
DIDOPO	364.98	425.28	1.537	—	—
PET/7.5 wt% DIDOPO	375.78	437.52	9.364	10.51	−1.146
PET/10 wt% DIDOPO	376.02	436.58	9.952	10.27	−0.318
PET/12.5 wt% DIDOPO	378.24	435.64	11.96	10.03	1.93
PET/15 wt% DIDOPO	377.25	436.15	14.61	9.78	4.83

### 3.4 Analysis of gas phase products in thermal degradation of PET/DIDOPO conjugated flame retardant composites

According to the flame retardant test results, it can be seen that DIDOPO has better flame retardant performance in PET, but its char-forming effect is relatively weak. It is speculated that the composite material has better flame retardant performance mainly due to the gas-phase flame retardant effect. To further analyze and confirm the gas-phase flame retardant effect of DIDOPO, a TG-IR test has been performed on DIDOPO and PET/DIDOPO to study the thermal degradation of gas-phase products. Figures 2C,D shows the TG-IR curves of DIDOPO and PET/DIDOPO at different thermal degradation temperatures. For PET/DIDOPO, gas-phase products began to be formed at 350°C, which was earlier than that of PET, indicating that DIDOPO promoted the degradation of PET, which was consistent with the TG test results. It can be seen from Figure 2 that the characteristic peaks of the flame retardant DIDOPO are mainly concentrated in the region of 700 cm^−1^ to 1,500 cm^−1^, and the peak intensity is the highest when the temperature is 438°C. Among them, 1,448 cm^−1^ corresponds to the P-O-CAr group, 906 cm^−1^ and 1,213 cm^−1^ correspond to the characteristic peaks of the P = O group, and 1,112 cm^−1^ corresponds to the absorption peak of P-O, and 749 cm^−1^ corresponds to the absorption of the P-C bond peak (Wang et al., 2001; Perret et al., 2011; Bai et al., 2014; Liu and Yao, 2017). This indicates that phosphorus-containing products with P-C, P = O and P-O bonds are generated during the thermal degradation of DIDOPO, and the thermal decomposition and dehydration processes of its phosphorus-containing groups induced the gases release e.g., H2O and CO/CO2, which can dilute the concentration of oxygen (Cao et al., 2022a). Besides, PET/DIDOPO has many characteristic peaks of phosphorus-containing substances at 700 cm^−1^∼1500 cm^−1^. It can be seen that DIDOPO will generate a large number of phosphorus-containing free radicals during the degradation process, and these free radicals will capture free radicals (e.g.,O·and H radicals) in the gas phase, which can reduce the amount of combustible products and avoid the spread of combustion reactions (Cao et al., 2022b; Mu et al., 2022).

## 4 Conclusion

In this paper, DIDOPO was used as a flame retardant for PET to investigate the effects of DIDOPO on the thermal degradation behavior, flame retardancy, and rheological behavior of PET materials. The results show that only adding 12.5% of DIDOPO can make the flame retardant grade of the composite material reach the UL-94 V-0 level, and the LOI value is increased by 56.87% compared with PET. The thermal degradation behavior and TG-IR analysis show that DIDOPO promote the degradation of PET, leading to a slight increase of char residue. The result suggests that DIDOPO mainly exhibited a gas-phase flame retardant effect, accompanied by a weaker solid-phase flame retardant effect. When the PET/DIDOPO composite is heated, phosphorus-containing free radicals and incombustible gases of DOPO derivatives will be generated, which will react with the free radicals generated during the combustion process of PET, relieving the progress of the combustion. Besides, the addition of DIDOPO can reduce the melt viscosity of the composite material. Accordingly, the melt droplets increase, and these droplets can also take part of the flame and heat away, improving the flame retardant properties of the composite material.

## Data Availability

The original contributions presented in the study are included in the article/Supplementary Material, further inquiries can be directed to the corresponding authors.
